# Effectiveness of a behavioral incentive scheme linked to goal achievement: study protocol for a randomized controlled trial

**DOI:** 10.1186/s13063-016-1161-3

**Published:** 2016-01-16

**Authors:** Julie Redfern, Gemma Enright, Simon Raadsma, Margaret Allman-Farinelli, Christine Innes-Hughes, Santash Khanal, Sarah Lukeis, Chris Rissel, Alex Gyani

**Affiliations:** The George Institute for Global Health, Sydney Medical School, University of Sydney, Level 10, King George V Building, Missenden Road, Camperdown, Sydney, NSW Australia; Department of Premier and Cabinet, Sydney, Australia; University of Sydney, Sydney, Australia; NSW Office of Preventive Health, Ministry of Health, Sydney, Australia; Better Health Company, Melbourne, Australia

**Keywords:** Prevention, obesity, health services, incentives, behavior change, overweight, children

## Abstract

**Background:**

Childhood obesity is a concern in Australia and across the world. Community-based weight management programs are an important response to address childhood obesity. However, the scientific literature suggests that their effectiveness could potentially be enhanced by providing a structured incentive scheme. This proposal aims to determine the effectiveness of enhanced goal setting linked to a structured incentive scheme designed to improve the sustained health and wellbeing of overweight/obese children within the context of an existing community-based program.

**Methods/Design:**

This study is a cluster randomized controlled trial delivered within the context of the existing NSW “Go4Fun” program with a 10-week and 6- and 12-month follow-up (*n* = 40 sites, 570 participants) that compares the effectiveness of small changes to the program in which children were asked to set goals (supported by text messages) and were given rewards for achieving them (intervention). This will be compared to the standard/existing program (control), which did not have the same structured incentive program. Data will be collected for all participants at baseline, end of program, and at 6 and 12 months. The primary outcome is a mean change in body mass index (BMI) *z* score at the 12-month follow-up. Secondary outcomes include anthropometric measures (body weight, height, and waist circumference) and behavioral measures collected via validated questionnaires. A process evaluation (comprising surveys and focus groups) to determine acceptability and sustainability and to inform downstream translation will also be conducted.

**Discussion:**

This study will inform policy and program delivery as well as the broader evidence base regarding goal achievement and incentive schemes directed at children’s health-related behaviors and will provide evidence that is likely to be transferrable across a range of health conditions.

**Trial Registration:**

ACTRN12615000558527 registered on 29 May 2015.

## Background

### Childhood obesity problem definition

The prevalence of childhood overweight and obesity is increasing, contributes to a major public health problem, and has implications for current and future health services [1]. Results from the 2011/12 Australian Health Survey showed that one-quarter of all Australian children (around 600,000 children) aged 5 to 17 years were overweight or obese, up four percentage points from 1995 (21 %) [2]. The extent of the epidemic and its short- and long-term effects on the physical and psychological health (including a potential reduction in life expectancy for future generations) have made the prevention and treatment of childhood overweight and obesity a high priority [3]. Importantly, being obese or overweight as a child increases the risk of a range of noncommunicable (or chronic) diseases and disorders in adulthood such as type 2 diabetes and cardiovascular disease [4]. Obese children have a 50 % chance of being overweight or obese as adults, and adults who were overweight as children have higher risk of weight related ill health and early death than people who became obese in adulthood [1]. In addition, being overweight or obese as a child can impact self-esteem, willingness to participate in class/sporting activities, and socialization. Despite the high prevalence and existence of multiple national action strategies and guidelines, child-focused obesity services are limited and opportunities exist for optimizing health-related behaviors [5, 6].

### Targeted programs to address childhood obesity

Community-based weight management programs are an important response to address childhood overweight and obesity. International recommendations identify that the core elements of any initiative to address the widespread problem should involve the family and include nutrition education, promote physical activity and include strategies that target behavior modification [2, 7–9]. Two recent Australian randomized controlled trials (RCTs), namely PEACH (*n* = 169) [10] and HIKCUPS (*n* = 165) [11] highlight the importance of engaging and targeting parents in structured weight loss programs for overweight/obese children aged 5 to 9 years old. Another RCT (*n* = 111) found that a parent-directed approach based on parenting skills training with lifestyle education improved BMI z scores by approximately 10 % compared to approximately 5 % with parenting-skills training alone or wait-listing for intervention [12]. This study demonstrates the importance of including parents in strategies aimed at improving weight management in children. Although, the specific behaviors required for effective weight loss are well established, the challenges associated with the behavior change aspects of interventions are profound; therefore, for these and many other reasons, trials are often not upscaled and translated into a real-world context [9, 13, 14].

The United Kingdom Mind Exercise Nutrition Do it (UK MEND) program is an evidence-based community-based child weight management program with effectiveness in weight outcomes [15, 16]. The MEND RCT was designed to assess effectiveness of a 9-week intervention based on the principles of psychology, learning, and social cognitive theories. The MEND trial (n = 117) demonstrated that the intervention group had significantly reduced waist and BMI measures, as well as improvements in physical activity and self-esteem [16]. Based on these findings, and due to the growing burden of childhood obesity in New South Wales (NSW, Australia), MEND was translated into the NSW Health context in 2009. The program was named Go4Fun and places an emphasis on reaching disadvantaged communities and, accordingly, low socioeconomic and regional areas [17]. It is a community-based, multidisciplinary family-focused program that targets weight-related behaviors and self-esteem of children aged 7 to 13 years who are overweight or obese [18]. To participate, children must have a BMI > 85th percentile for their age and sex (as per the Australian Institute of Health and Welfare (AIHW) classification of overweight and obesity in children) [19]. The program is centrally managed by the NSW Office of Preventive Health, with NSW local health districts (LHDs) and the Better Health Company being responsible for delivery of the 10-week program. In line with current evidence about the importance of family engagement, both the parent/guardian and the child attend the weekly sessions.

While Go4Fun has demonstrated short- and medium-term health benefits for those who complete it, opportunities to improve retention and completion, goal setting and outcomes, and sustained outcomes after the program have been identified. A recent process evaluation found that despite the potential as a community-based population-wide program, widespread reach and attendance could be improved [17]. End of program results (10 weeks) are encouraging, with participating children achieving a mean reduction in BMI (0.7 kg/m^2^) and waist circumference (1.7 cm) as well as improvements in physical activity and self-esteem [18]. Some Go4Fun local program providers have tried to improve retention and goal achievement through the ad hoc use of incentives (such as stickers and loyalty cards and in some cases, vouchers), but these incentives are not standardized; nor have they been investigated in isolation. While a quality improvement framework is in place, great value exists in robustly evaluating whether or not a program can be improved by incentivization. This concept is supported by recent literature emphasizing the value of evaluation in policy and service improvement, where incremental changes supported by small scale and tightly focused approaches can lead to scalable impacts over time [20]. Therefore, research is needed to maximize the reach and value of Go4Fun and other weight management programs. One new approach is to consider behavioral and psychological literature relating to the use of incentives to support behavior change through goal setting.

### Behavioral insights literature

Challenges in facilitating behavior change, as it relates to human health, has led to increasing prominence of research investigating whether the use of incentive schemes (based on psychological and behavioral theory and research) might positively influence health-related behaviors [21]. As such, researchers have explored the potential value of incentives in the formation of exercise habits [22], weight loss [23, 24], and smoking cessation [25]. Two recent systematic reviews reporting studies in adults found interventions based on providing financial incentives are more effective than usual care for encouraging healthy behavior change [26] and that lucky draw style rewards were an effective mechanism for upscaling [26, 27]. The reviews also found that making rewards contingent on repeated behaviors maximised habit formation and sustainability [27]. Further research in adults has found that rewards for goal attainment (for example, the completion of daily walking) were more likely to have positive longer-term outcomes than simply rewarding outcomes (such as losing a certain number of kilograms) [28, 29]. Other studies have also demonstrated that treatments that focus on behaviors, rather than outcomes have been effective in numerous meta-analyses to change behavior in general [30] and for promoting physical activity [31]. In addition, evidence suggests that the use of small rewards can increase task perseverance as people are motivated to complete an action when they can see their progress [32].

Despite encouraging developments in adults, to date there has been very little research investigating the value of incentives for improving health-related behaviors in children. One French nonrandomized study (*n* = 1,589) investigated the effects of a school-based incentive program aimed at promoting physical activity [21]. Incentives were based on lottery style tickets earned by children when they participated in particular activities (for example, riding or walking to school) and they were entered into a regular prize draw ($10 sports store). The study found the incentive program increased the probability of physical activity and the authors conclude that the lucky draw aspect of the scheme provided a low-cost approach to promoting exercise in children [21]. Another RCT testing the effect of an incentive scheme aimed at improving fruit and vegetable intake by children found that at baseline 33 % of children ate at least one serving of fruit or vegetables during the school day and at follow-up this increased to an average of 60 % in the incentive group and remained unchanged (31 %) in the control group [33]. The authors also tested the difference between different reward schemes and found that immediate provision of rewards when goals were achieved resulted in a larger effect than delayed provision. Further insights can be gleaned from nonhealth related fields. A recent paper collates the findings of a series of school-based RCTs in more than 200 American urban schools testing the impact of financial incentives on student’s reading books and their reading achievement. This study found that incentives were more effective when they were given to children when they performed a behavior (in this case reading) rather than for achievement of an outcome (in this case exam results). Whilst this study has substantial limitations (for example, a lack of comparability across samples) it adds weight to the speculation on the impact of incentives in the achievement of behavioral goals (inputs) rather than health outcomes (outputs) in school-aged children [34].

The issue of sustainability of behavior, as it relates to incentives, is also an important area of consideration. Evidence suggests that incentivising activities and goals is more likely to have a long-term outcome than simply incentivising outcomes, which tends to have a powerful but often transient effect [32]. The theoretical concept is that incentives directed at activities and goals facilitates creation of behavioral habits that improve sustainability. However, there is very limited research that has tested retention of behavior change after incentives are ceased. Another area of research that has been shown to improve retention of health-related behaviors is the use of ongoing and regular text messages. These include RCTs demonstrating effectiveness of mobile phone text messaging to promote smoking cessation [35, 36], weight loss [37], physical activity [38], asthma medication adherence [39], and glycemic control in diabetes [40]. Further studies have examined the effects of text message reminders and support on medication adherence. The largest of these (538 participants) delivered text messages with health counsellor support over 12 months to HIV patients. Patients receiving the intervention had significantly improved adherence to antiretroviral treatment and rates of viral suppression compared with control individuals [41]. Other smaller studies have found improved self-efficacy and medication adherence for patients with diabetes [40] and asthma [39] and in pediatric transplant patients [42].

In summary, early research suggests that the use of incentives for promoting behavior change in overweight/obese children has potential, but to date, this is a relatively unexplored area. The overall objective of this research is to apply behavioral insights to determine the effectiveness of a structured goal setting and incentive scheme to improve health-related behaviors in overweight/obese children. The opportunity also exists to test, using text message reminders and lottery prize draw, whether any gains made during the program are maintained and whether the effects of incentives are sustained after the program, as a cost-effective way of engaging with families after the program. The specific aims of this research are as follows:

1. To determine the effectiveness of a structured goal setting incentive scheme on the following:
  i. Health outcomes (body mass index (BMI) and waist *z* scores, BMI, waist circumference, nutrition, physical activity, and self-esteem) at the end of the program (10 weeks), 6 months, and 12 months;
  ii. Completion of at least 75 % of the Go4Fun weight management program and;
  iii. Achievement of physical activity and nutrition goals during the Go4Fun program.
2. To determine the effectiveness of post program text message reminders highlighting a lottery prize draw on the following:
  i. Sustained health outcomes (BMI and waist *z* scores, BMI, waist circumference, nutrition, physical activity, and self-esteem) at 6 months, and 12 months after the program; and
  ii. Achievement of physical activity and nutrition goals at the end of the program (10 weeks), 6 months, and 12 months after the program.
3. To determine the acceptability (via a process evaluation) of the structured incentive scheme to inform widespread policy translation and implementation.

## Methods/Design

### Study design

**Fig. 1:**
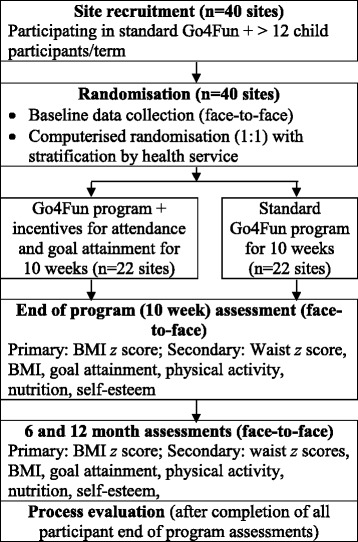
Behavioral incentive study schema. BMI, body mass index; QOL, quality of life

The study is a cluster RCT (Fig. 1) delivered within the context of the existing Go4Fun program with 10 week, 6 month, and 12 month follow-up (*n* = 40 sites, 570 participants), which compares the effectiveness of participation in the standard Go4Fun program with an enhanced goal setting and structured incentive scheme (intervention) versus participation in the existing Go4Fun program (control). Individual randomization is not possible given the nature of the intervention, so randomization by site will be conducted. Building on our existing research platform, we propose a pragmatic and feasible trial to determine the effectiveness, acceptability, and sustainability of the enhanced intervention at 6 and 12 months after the program. The CONSORT statements for cluster RCTs [43] and for nonpharmacological interventions [44] will be followed.

### Eligibility/recruitment: Sites (namely programs where Go4Fun is delivered)

To be eligible to participate in the trial, sites must meet the following criteria:

1. Are delivering the standard Go4Fun program in 2015;
2. Show a 2014 attendance average of at least 10 children enrolled per program per term; and
3. Are willing to participate in a trial and adhere to standardized procedures for the duration of the trial.

For site recruitment, all NSW LHDs (where Go4Fun is delivered) were invited to participate, 40 programs agreed to participate, and ethical approval was secured from the relevant LHDs. Based on prior process evaluation work [17] and from prior attendance records, the recruited sites are likely to enroll an average of 12 to 15 children per site per term. Local program leaders will assess eligibility, obtain informed consent, and conduct baseline study visits, and research assistants will conduct the follow-up assessments. Previous RCTs conducted by the research team have underscored the critical importance of site-based program leader involvement and engagement to maximize downstream sustainability.

### Eligibility/recruitment: Participants

To be eligible to participate in this trial, leaders were instructed that children must meet the following criteria:

i. Aged 7 to 13 years;
ii. Have a body mass index > 85th percentile for their age and sex (according to AIHW classification of overweight/obesity in children) [19, 45];
iii. Be enrolled in and meet the general criteria to participate in the Go4Fun program at one of the sites participating in this study, which includes having a parent or adult carer able to accompany them to each session; and
iv. Parent/guardian provides written and informed consent.

Participant/child eligibility for the Go4Fun program is assessed at the time of referral/contact with their LHD. No changes will be made to this standard program enrolment process. Eligibility will be determined by the program leaders based on assessment of the above criteria once the child attends the initial intake appointment, and written informed consent will be sought prior to collection of baseline data.

### Randomization

Eligible sites have been randomized to either deliver the intervention (standard Go4Fun plus incentives) or control (standard Go4Fun) program for 10 weeks. Randomization was conducted using a computer-generated sequence (1:1) managed by the research team based at The George Institute and independent of the Department of Premier and Cabinet NSW (DPC), any program leader or LHD. Clusters were stratified according to local health district to ensure equal representation across groups within each LHD.

### Development of incentive scheme and standardized program

In late 2014, an iterative process combining literature review, focus groups, and consensus meetings took place with three aims: namely, to (i) identify the best possible incentive scheme based on available literature, (ii) standardize the existing Go4Fun attendance rewards system across all treatment and control sites, and (iii) develop and agree on the goal setting scheme and incentive structure to be used for the intervention group. Participants included representatives from authorship group, program leaders representing all LHDs, DPC (the Government department responsible for delivery of Government priorities in NSW, Australia), Better Health Company, and the NSW Office of Preventive Health. In addition, investigators from DPC conducted six field research visits (including interviews) to collect feedback and ideas from the Go4Fun leaders about potential ways to enhance the goal setting process and practical considerations regarding delivery and impact as well as post-program engagement opportunities. A literature review demonstrated the importance of incentivizing behavior rather than outcomes. This approach was confirmed by Go4Fun leaders during early fieldwork so that, despite being a core part of the Go4Fun program, goal-setting structures could be improved by making the goals specific, measureable, achievable, relevant, and timely (SMART) [32]. This emphasized the importance of enhancing the goal-setting process, including resetting/stretching them if they are achieved too easily in the program, as well as linking goal achievement to incentives [32].

Preliminary work highlighted the need for a standardized reward system for the control program, and “healthy,” positive, and low-cost incentives (for example, items that encourage physical activity and nutrition) were needed for the intervention group. It was also agreed that incentives should be of increasing value as they become harder to achieve. Once consensus was reached regarding the control program and the incentive scheme for the intervention, formal standard operating procedures were prepared and circulated for revision and refinement until consensus was reached. This process was important to facilitate engagement and satisfaction of site leaders and to minimize potential variability between sites. Specific rewards and associated delivery structure are outlined further in the “intervention” section of this proposal.

### Control group

Sites randomly allocated to the control group will continue to deliver the standard Go4Fun program. As described above, the standard program consists of weekly group sessions (one per week) for 10 weeks during the school term. Exercise sessions involve one hour of activities that progressively develop skills, strength, fitness, confidence and self-esteem [17]. Nutrition sessions include healthy eating advice, food label reading and recipes [17]. Behavioral change sessions include goal setting, problem solving and role modelling to influence habits and behavior around eating and exercise [17]. Through preliminary focus groups, consensus was reached to ensure the standardization of Go4Fun between sites. In particular, when and how children receive reward “stickers” was standardized throughout the control program. It was also agreed that children would receive incentives such as water bottles (for attending one session), bouncy balls (for attending three sessions), and skipping ropes (for attending 10 sessions) based on their record of attendance.

### Intervention group

Sites randomly allocated to the intervention arm will deliver the Go4Fun program with the enhanced goal setting and structured incentive scheme developed during our preliminary work. As described above, the standard program consists of weekly group sessions (one per week) for 10 weeks during the school term. No sessions have been lengthened or combined for the intervention sites. All children participating in the intervention sites will receive the same information and participate in the same exercise and nutrition sessions as the control group. They will also receive the same system of “reward stickers” and standardized attendance rewards throughout the program. However, at intervention sites, participating children will be eligible to participate in an enhanced goal-setting component and receive incentives for reaching certain levels of goal attainment. That is, for the intervention, participants will set SMART [32] behavioral goals and achieving these goals will result in the incentives being provided. This emphasizes the importance of enhancing the goal-setting process, including resetting/stretching them if they are achieved too easily in the program, as well as linking goal achievement to incentives [32]. The incentives will only be received for the period of the 10-week program.

The goal setting component and incentive scheme was developed and agreed upon during the preliminary work for this study with an overview of the goal setting enhancement and incentives being as follows:

1. *Goal setting:* At the third session in the program, the children and their parent/ guardian in the intervention group are provided with an enhanced resource to guide them through jointly setting an exercise and a nutrition goal (child and parent/guardian in collaboration). Examples include ‘I will play soccer for 30 minutes on 3 days a week at the park with dad’ and ‘I will try a new vegetable two times a week for dinner on Wednesday and Sunday nights.’
2. *Goal attainment incentives:* Children will receive milestone-based incentives for achieving their set goals. There will be three levels as follows: (i) a vegetable slicer once two exercise and two nutrition goals are achieved; (ii) a sports store voucher (value of $AUS10) once four exercise and four nutrition goals are achieved, and (iii) a height-adjustable tennis set once six exercise and six nutrition goals are achieved. In addition, Go4Fun leaders will ask the children on a weekly basis if they have achieved their goal and prompt them to reset them if they were too easy.
3. *Goal attainment reminders via text message and lottery style incentive:* After the 10-week program, parents/ guardians will receive weekly mobile phone text message reminders to support and encourage children to achieve their goals (and set new ones where relevant). Parents/ guardians will be encouraged to text back with goal achievements with each goal achieved being rewarded with a ticket entry (maximum of eight tickets per month) into a prize draw (one per program) for a family pass to a local zoo (or similar family activity, depending on local availability) that will be drawn at 6 months.

### Outcomes

Data will be collected for all participants at baseline, end of program, at 6 months, and 12 months. The primary outcome is a mean change in BMI *z* score at 12-month follow-up. BMI *z* scores indicate how many units (of the standard deviation) a child’s BMI is above or below the average BMI value for their age and sex. BMI *z* scores will be calculated from raw BMI measures using the Centers for Disease Control growth reference data [46]. Secondary outcomes include anthropometric measures (body weight, height, and waist circumference) assessed according to standardized procedures [47] and behavioral measures collected via validated questionnaires (Table 1). Similar to the BMI *z* scores, waist measures in centimeters will be converted to a waist circumference *z* score based on reference data [46]. The Physical Activity Enjoyment Scale (PACES) will be used to assess physical activity, and this tool has been found to have good internal consistency, item-total correlations, and validity in primary school children (*n* = 564) [48]. Scores were also correlated with task goal orientation, athletic competence, physical appearance and self-reported physical activity [48]. For nutrition questions, we selected relevant questions from the NSW Centre for Public Health Nutrition recommendations for nutrition questions [49]. These questions are recommended for practical assessment and have been developed based on evidence and guidelines for children [49]. An adapted version of the Rosenberg Self-Esteem Scale will be used and is one of the most widely used self-esteem measures in social science research [50]. This scale has been tested for reliability and validity in many languages and has been found to be effective including in children [50].

**Table 1:** Primary and secondary outcomes (measured at baseline, end of program, 6 months, and 12 months)

Primary
• Mean change in BMI *z* score* (measured using standardized procedures [47]) at the end of the program (10 weeks) and at the 6-month and 12-month follow-up
Secondary (measured at the end of the program and at the 6-month and 12-month follow-up)
• Mean change in mean waist circumference and waist *z* score, measured using standardized procedures [47]
• Mean change in mean BMI* - measured using standardized procedures [47]
• Difference in mean rate of goal attainment between intervention and control groups for nutrition and physical activity – child diaries verified by parent/guardian
• Mean difference in physical activity level – Physical Activity Enjoyment Scale (PACES) [48]
• Difference in fruit and vegetable, breads and cereals and dairy intake per day; fast food and sweetened drink intake per week - based on NSW Centre for Public Health Nutrition recommendations for nutrition questions [49]
• Mean difference self-esteem – Rosenberg Self-esteem Scale [50]

*BMI (kg/m^2^) calculated based on the measurement of height and weight using standardized procedures [47] and BMI *z* score calculated based on Centers for Disease Control reference data [46]

### Data collection

Data will be collected at baseline, end of program, 6 months, and 12 months by research assistants. For baseline measures, staff members have been trained by the Better Health Company investigator team in measuring height, weight, and waist circumference using standardized procedures [47]. All participants will be contacted to attend the follow-up sessions, and they will be incentivized with a gift voucher (value of $AUS25) for attending each of the 6- and 12-month health checks These payments are to maximize the rate of follow-up for the research study and are offered to both control and intervention site participants. Data will be entered into a secure online database that will require minor additional functionality for the collection of goal attainment and 6- and 12-month follow-up data.

### Sample size

Intraclass correlation was calculated based on preliminary data (214 individuals) across the recruited 40 sites and was found to be 0.16 for the BMI *z* score. To detect a between-group difference of 0.24 (±0.43) in the BMI *z* score (based on outcome data from a previous Australian RCT examining 12-month weight loss outcomes in children) [12], 12 participants from each of the 40 sites (20 intervention, 20 control) are required to achieve 80 % power based on an alpha of 0.05.

### Statistical analysis

Analysis will be conducted at the individual level and will follow the intention-to-treat principle. The control and intervention groups will be compared on baseline characteristics using independent t-tests for continuous variables or chi square tests for categorical variables. For the primary outcome, the mean difference in BMI *z* score at 12 months between control and intervention groups will be analyzed after adjusting for the baseline characteristics using regression models. Similar regression models will be used for the secondary outcomes. These models will account for the between-cluster variance and will be adjusted for baseline characteristics. All analyses will be undertaken using SAS 9.4 for Windows (SAS Institute Inc. Cary, NC, USA), and statistical significance will be set at *P* < 0.05.

### Process evaluation

Process evaluations explore the implementation, receipt, and setting of an intervention and help in the interpretation of outcome results [51]. Information generated from the process evaluation will inform downstream translation by informing policymakers and program developers about the intervention strengths, weaknesses, and areas for improvement. We will use a mixed methods approach to investigate why the incentive scheme may or may not have been effective/sustainable and which intervention components were most influential. Four data sources will be used: (i) quantitative data related to “reach” on participant attendance and outcomes described above; (ii) for those in intervention group, participant and parent/guardian surveys to examine intervention value, satisfaction, utility and health behaviors influenced; (ii) semi-structured interviews with Go4Fun program leaders to investigate implementation along with associated barriers and enablers; and (iv) focus groups with participants and parents/guardians to explore uptake, acceptability and suggestions for improvement. Taken together, qualitative data will explore participant and program leader views on benefits, disadvantages, acceptability of and potential improvements for the incentive scheme. To obtain a broad range of views, we will use a maximum variation sampling method based on patient demographics and health service characteristics [52]. Sampling will continue until no new themes or categories emerge (“thematic saturation”). Analyses will be thematic, and coding will be carried out inductively based on emergent themes. NVivo 9 will be used to assist with interview data management. 

### Ethical approval

This study will adhere to the Australian National Health and Medical Research Council ethical guidelines for human research. Lead ethical approval has been obtained from the Sydney South Western LHD Research and Ethics Office (HREC/13/LPOOL/157), and necessary governance clearances have been approved as follows: South West Sydney Human Ethics Committee: HREC/14/LPOOL/480 and local project number: 14/278; South Eastern Sydney Local Health District Research Governance: SSA/14/G/398; Western Sydney Local Health District Research Governance: SSA/15/WMEAD/ 40; Hunter New England Local Health District Research Governance: SSA/15/HNE/110; Northern Sydney Local Health District Research Governance: SSA/15/HAWKE/110; and North Coast Local Health District Research Governance: SSA/14/NCC/126. Written informed consent will be obtained from all child participants and their parent/guardian prior to their participation. Participants will be free to withdraw at any time.

## Discussion

Scientific literature suggests that providing an incentive scheme linked to goal achievement may improve motivation and completion of behavior change programs, but the potential of such schemes and their optimal delivery remain relatively unexplored in children. This study will inform policy and program delivery as well as the broader evidence base regarding goal achievement and incentive schemes directed at children’s health-related behaviors that will provide evidence that is likely to be transferrable across a range of health conditions.

If the intervention is effective, it is likely to improve behavior change and enhance the outcomes for participants, thus reducing morbidity and mortality as well as the incidence of heart and cardiovascular disease in the future. The intervention aims to target prevention through incentivization of improved behavior change in a high-risk population of children. It is of great significance that the study is being conducted by a collaborative team that includes research leaders, behavioral change experts, and policymakers who are responsible for delivery of the government-funded weight management program. Ultimately, this will facilitate downstream translation and make a more effective program available to more high-risk children.

## Trial status

As at 1 July 2015, recruitment has commenced and follow-up assessments are ongoing. No data cleaning or analysis of results had begun at the time of this submission.

## Abbreviations

AIHW: Australian Institute of Health and Welfare
BMI: body mass index
CONSORT: Consolidated Standards of Reporting Trials
DPC: Department of Premier and Cabinet
HIV: Human Immunodeficiency Virus
LHDs: Local Health Districts
MEND: Mind Exercise Nutrition Do it
NSW: New South Wales
RCT: randomized controlled trials
SMART: specific, measureable, achievable, relevant, and timely
UK: United Kingdom
