# Erratum to: ‘Effectiveness of a behavioral incentive scheme linked to goal achievement: study protocol for a randomized controlled trial’

**DOI:** 10.1186/s13063-016-1254-z

**Published:** 2016-03-08

**Authors:** Julie Redfern, Gemma Enright, Simon Raadsma, Margaret Allman-Farinelli, Christine Innes-Hughes, Santosh Khanal, Sarah Lukeis, Chris Rissel, Alex Gyani

**Affiliations:** The George Institute for Global Health, Sydney Medical School, University of Sydney, Level 10, King George V Building, Missenden Road, Camperdown, Sydney, NSW Australia; Department of Premier and Cabinet, Sydney, Australia; University of Sydney, Sydney, Australia; NSW Office of Preventive Health, Ministry of Health, Sydney, Australia; Better Health Company, Melbourne, Australia

Unfortunately, the original version of this article [1] contained an error. An author’s name was spelt incorrectly. Santosh Khanal is the correct spelling of the author’s name and is included here. There will also be an update in addition to this erratum for this change.
